# The *DROOPING LEAF* (*DR*) gene encoding GDSL esterase is involved in silica deposition in rice (*Oryza sativa* L.)

**DOI:** 10.1371/journal.pone.0238887

**Published:** 2020-09-10

**Authors:** Yoye Yu, Mi-Ok Woo, Piao Rihua, Hee-Jong Koh

**Affiliations:** 1 Department of Plant Science and Research Institute for Agriculture and Life Sciences, and Plant Genomics and Breeding Institute, Seoul National University, Seoul, South Korea; 2 Science & Technology Policy Division, Ministry of Agriculture, Food and Rural Affairs, Sejong, South Korea; 3 Rice Research Institute, Jilin Academy of Agricultural Sciences, Gongzhuling, Jilin, China; Kyung Hee Univeristy, REPUBLIC OF KOREA

## Abstract

Leaf morphology is one of the most important agronomic traits in rice breeding because of its contribution to crop yield. The drooping leaf (*dr*) mutant was developed from the Ilpum rice cultivar by ethyl methanesulfonate (EMS) mutagenesis. Compared with the wild type, *dr* plants exhibited drooping leaves accompanied by a small midrib, short panicle, and reduced plant height. The phenotype of the *dr* plant was caused by a mutation within a single recessive gene on chromosome 2, *dr* (LOC_Os02g15230), which encodes a GDSL esterase. Analysis of wild-type and *dr* sequences revealed that the *dr* allele carried a single nucleotide substitution, glycine to aspartic acid. RNAi targeted to LOC_Os02g15230 produced same phenotypes to the *dr* mutation, confirming LOC_Os02g15230 as the *dr* gene. Microscopic observations and plant nutrient analysis of SiO_2_ revealed that silica was less abundant in *dr* leaves than in wild-type leaves. This study suggests that the *dr* gene is involved in the regulation of silica deposition and that disruption of silica processes lead to drooping leaf phenotypes.

## Introduction

Plant architecture has substantial impacts on growth and yield as a consequence of its direct influence on photosynthesis. Erect or excessively curved leaves increase plant self-shading, thereby decreasing light interception and decreasing photosynthetic capacity. Optimal leaf shape is therefore a critical characteristic that impacts a range of physiological functions. Enhancing leaf morphology, such as by reducing leaf drooping, is a key aim of plant breeding programs [1].

Many factors contribute to drooping leaf morphology, such as lack of midribs, abnormal arrangement of bulliform cells, modulated wax synthesis, lignin content, and silica presence. Midribs, which keep leaves upright, are thickened by cell proliferation, and promotion of cell proliferation impacts leaf phenotype [2]. Bulliform cells are found between two vascular bundles in parallel on the adaxial side of leaves. In a previous study, Xu et al. [3] reported that increased numbers and abnormal arrangements of bulliform cells increased leaf distortion and contributed to leaf drooping. Leaf surfaces are covered by a dense layer of wax crystals and loosening of layers leads to reduced wax deposition and distortion of leaf shape [4]. Lignin and silica can form tight crystal complexes with cellulose in secondary cell walls, generating layers of up to 2.5 μm in thickness. Reductions in lignin and silica accumulation decrease cell wall robustness and thereby influence leaf phenotype [5, 6].

Several genes related to the drooping leaf phenotype have been cloned and analyzed in rice. The *DL* and *dl2* genes, which are involved in midrib formation, are located on chromosomes 3 and 1, respectively, and encode YABBY domains [7, 8]. Abnormal bulliform cell arrangement is regulated by the *OsZHD1* and *OsZHD2* genes, which are located on chromosomes 9 and 8, respectively. *OsZHD1* and *OsZHD2* encode zinc finger homeodomain class homeobox transcription factors [3]. The *OsWR1* gene on chromosome 2 encodes ERF proteins and controls wax synthesis through alteration of long chain fatty acids and alkanes [9]. Secondary cell wall and lignin formation are regulated by the *OsSWN1* gene, which is located on chromosome 6 and encodes a NAC transcription factor [10, 11]. Several silica transporter genes, *Lsi1*, *Lsi2*, and *Lsi6*, located on chromosomes 2, 3, and 6, respectively, encode aquaporin-like proteins [12–14].

In this study, whole genome sequencing and MutMap analysis was used to investigate a novel rice mutant gene, drooping leaf (*dr*). The *dr* gene was cloned and validated using RNAi. Transmission and scanning electron microscopy (TEM and SEM) and silica content analyses were used to further understand the cause underlying leaf shape determination.

## Material and methods

### Plant materials

Ethyl methanesulfonate (EMS) treatment was used to induce the *dr* mutation in a Korean *japonica* rice cultivar, Ilpum. Seeds of the mutant line used in this study were taken from the M_13_ generation. An F_2_ population derived from a cross between the mutant and the wild-type was used for genetic analysis and whole genome sequencing. For genetic mapping, Milyang 23, a Korean *Tongil*-type rice cultivar, was crossed with the mutant to obtain the mapping population. All plant materials were grown at the Experimental Farm of Seoul National University (Suwon, Korea) using conventional cultural practices.

### Phenotype analysis

Agronomic traits including plant height (PH), stem length (SL), panicle length (PL), tillers per panicle (TP), panicles per plant (PP), spikelets per panicle (SP), spikelets per plant (SPP), and spikelet fertility (SF) were assessed in a single panicle from the main stem, with panicles assessed from 3–5 independent plants. Five replicates of thousand grain weights (TGW) were measured using an electronic balance (CAS, USA).

Chlorophyll (Chl) contents were measured in leaf sheaths from 70-day-old wild-type and mutant plants, using a Minolta SPAD-502 Meter (Minolta Camera Co., Ltd, Japan). Measurements were taken from five biological replicates.

For silica analysis, leaves were decolorized using 70% ethanol for 2 days at room temperature and then stained in phenol containing 0.001% safranin [15]. Photographs were taken under optical microscopy.

### Determination of SiO_2_, N, P, and K

Leaves, stems, and roots were harvested at the maximum tiller number stage. Three replicate samples were collected. Samples were dried at 105°C for 16 hours and then ground to a powder. Approximately 0.5 g powdered sample was mixed with 1 ml concentrated sulphuric acid (reagent grade) and 10 ml 50% perchloric acid in a Kjeldahl flask. The mixture was heated to 400°C for 3 hours, cooled, and then filtered into a 100 ml mass flask through No. 6 quantitative filter papers. Filtration residues were used for SiO_2_ analysis, and filtrates were diluted with distilled water for determination of N, P, and K.

### Histological analysis

Specimens for TEM and SEM were prepared from the maximum tillering stage leaves. For SEM, harvested leaves were fixed overnight at 4°C with slightly modified Karnovsky’s fixative consisting of 2% paraformaldehyde, 2% glutaraldehyde, and 50 mM sodium cacodylate buffer at pH 7.2, and then washed three times with 50 mM sodium cacodylate buffer. Samples were post-fixed with 1% osmium tetroxide in 50 mM sodium cacodylate buffer and then washed three times with distilled water. Samples were treated with 0.5% uranyl acetate, washed with an ethanol gradient series, and then treated with hexamethyldisilazane (HMDS). Samples were mounted on platinum stubs, coated with gold, and examined by a Field-Emission Scanning electron microscope (Sigma, Carl Zeiss).

TEM samples were fixed, post-fixed, and dehydrated as described in SEM, and then embedded in propylene oxide and Spurr’s resin overnight at 70°C. Embedded samples were sliced to 60 mm with an ultramicrotome (MT–X, RMC), and then stained with 2% uranyl acetate for 5 min and Reynold’s lead citrate for 2 min at 25°C. Processed samples were examined using a JEM-1010 EX electron microscope (JEOL, https://www.jeol.co.jp/en/).

### Genetic analysis and fine mapping of the *dr* gene

A total of 250 F_2_ progeny from a cross between the mutant and Milyang 23 was used for segregation analysis and gene mapping. Genomic DNA samples were extracted from rice leaves using the CTAB method [16]. A bulked segregant analysis (BSA) strategy was used for genetic mapping as described by Michelmore et al. [17]. Equal amounts of DNA from each of ten mutant and ten wild-type plants were pooled into single bulked samples. Sixty-five sequence-tagged site (STS) markers designed at the Crop Molecular Breeding Laboratory, Seoul National University, were used to analyze bulk samples. The STS markers were distributed throughout the rice genome at known chromosomal locations. After BSA, fine mapping was performed using seven additional STS markers designed against rice databases (http://www.gramene.org; https://rapdb.dna.affrc.go.jp) [18]. Polymerase chain reaction (PCR) was performed as described by Piao et al. with slight modifications [19]. Primer sequences are shown in S1 Table.

### Whole genome sequencing and MutMap analysis

DNA from 17 F_2_ plants displaying the *dr* phenotype were combined into a bulked sample for sequencing. Sequencing libraries were constructed from 5 μg bulked sample DNA using a TruseqNano DNA LT sample preparation kit (FC-121-4001). Libraries were used for cluster generation and were sequenced for 250 cycles on an Illumina HiSeq2500 platform. To generate the Ilpum reference sequence, 20.12 Gb of wild-type Ilpum sequence reads from KOBIC (www.kobic.re.kr, No. 2013-10000-3) was aligned to the Nipponbare reference genome (build five genome sequence; http://rapdblegacy.dna.affrc.go.jp/download/index.html) using BWA (Burrows-Wheeler Aligner) software. Alignment files were converted to SAM/BAM files using SAM tools, and the aligned short reads were filtered using Coval to improve SNP calling accuracy. The SNP index was calculated as described by Abe et al. [20]. Sliding window analysis was applied with a 4 Mb window size and a 10 kb increment following the improved MutMap methods [21, 22]. In the sliding window analysis, the average SNP index and average p-Value (Fisher’s exact test) were calculated for the SNPs located in the window.

### Derived CAPS (dCAPS) maker analysis

To test the co-segregation between SNP genotype of the mutation locus and mutant phenotype, dCAPS analysis was performed [18]. PCR amplification with the primer set Os02g15230-Tsp45I-F (5’- ACGCCTGTTATCCAGTTCA-3’) and Os02g15230-Tsp45I-R (5’- CATCACACGCGGTTGCACCAGCGT-3’) were performed. Primers were designed using the CAPS finder 2.0 program (http://helix.wustl.edu/dcaps/dcaps.html). Each product was digested with Tsp45I in a total volume of 15μl at 37°C for 3 hours. After digestion, 5μl of each digest was visualized by ethidium bromide staining on TBE gel.

### RNA isolation

Total RNA was isolated from the mutant and wild-type leaves using RNAiso plus (Takara Bio, Japan). Extracted RNA was treated with RNase-free Recombinant DNase Ι (Takara Bio, Japan) to eliminate gDNA contamination. Reverse transcription was performed using an M-MLV reverse transcriptase kit (Promega, Madison, WI, USA). For the amplification of full-length cDNA of the target gene, we designed a pair of primers based on the cDNA sequence of LOC_Os02g15230 from the rice database (https://rapdb.dna.affrc.go.jp), Os02g15230.1-full cDNA-F (5’-GGCGGCGATGGGGGCAGTTC-3’) which contains the 5’UTR sequence with start code and Os02g15230.1-full cDNA-R (5’-AAGTGACTTTCTCATGAAGA-3’) which was derived from 3’UTR sequences.

### Vector construction and rice transformation

The RNAi construct for *DR* gene suppression was amplified using the primers, Os02g15230-1-F (5’-AAAAAGCAGGCTACAAGCAGCTACCTAGGGCA-3’) and Os02g15230-1-R (5’-AGAAAGCTGGGTATACCCAACCTCTGGCAATG-3’), then the PCR products were cloned into pDONR201 (Invitrogen). Subsequently, entry clones with *DR* gene were inserted into pH7GWIWG2(II) and pHGWFS7, using Gateway^®^ BP and LR Clonase^™^ II enzyme mixes (Invitrogen, USA). The resultant constructs were introduced into Dongjin, a *japonica* rice cultivar, via Agrobacterium transformation using bacterial strain LBA4404. Transformation was performed as previously described with slight modifications [23].

### Multiple sequence alignment

The amino acid sequences of *DR* and other crops proteins were downloaded from NCBI (http://www.ncbi.nlm.nih.gov) and UniProt (http://www.uniprot.org) website. Multiple sequence alignments were conducted using Clustal Omega (https://www.ebi.ac.uk/Tools/msa/clustalo) and background color shading was applied with Jalview using the perentage identity scheme. The conserved domain prediction was performed using NCBI (http://www.ncbi.nlm.nih.gov/structure/cdd).

## Results

### Characterization of the *dr* mutant lines

**Fig 1:**
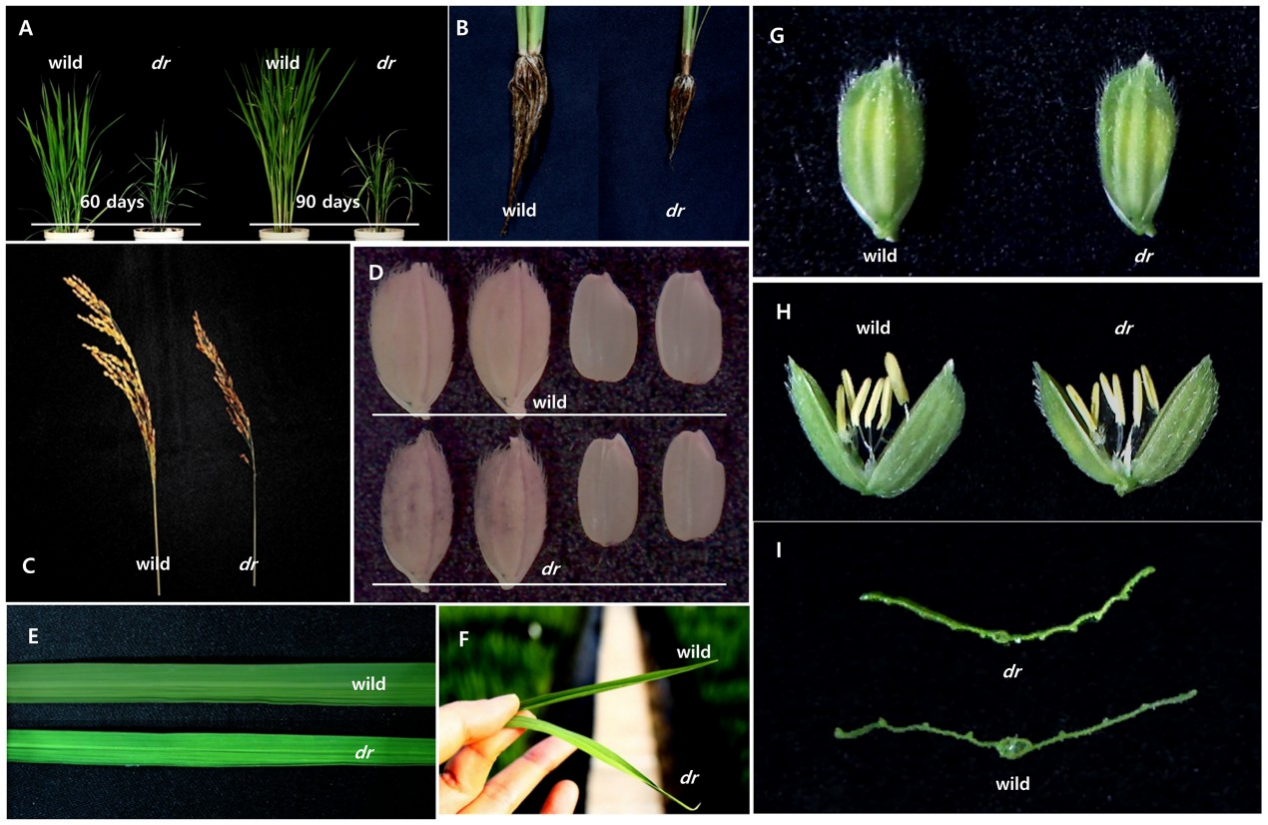
Comparison of the wild-type and the *dr* mutant phenotypes. (A) Overall shape of plants on 60-day-old (left) and 90-day-old(right). (B) The roots development in the wild-type (left) and the *dr* mutant (right). (C) Panicle shape of the wild-type (left) and the *dr* mutant (right). (D) Spikelets and seeds phenotypes of the wild-type (upper) and the *dr* mutant (down). (E) Morphology of 60-day-old leaves. (F) The degree of drooping leaves in the wild-type (upper) and the *dr* mutant (down). (G) Spikelets of wild-type (left) and the *dr* mutant (right) before flowering. (H) Physically opened spikelets of wild-type (left) and the *dr* mutant (right), before flowering. (I) Cross-section of the *dr* mutant (upper) and the wild-type (lower).

General agronomic traits were assessed to identify differences between wild-type and the *dr* mutant lines. Overall, the mutant exhibited significant stunting compared with the wild-type (Table 1). During the reproductive stage, the *dr* mutant exhibited short stem lengths that produced a substantial height reduction (Fig 1A) of approximately 50% compared with the wild-type. Numbers of lateral and adventitious roots were lower in the mutant (Fig 1B) compared with the wild-type, and primary roots were shorter. Panicle length, spikelet numbers per panicle, and spikelet numbers per plant were also lower in the mutant than in the wild-type (Fig 1C). Grains produced by the mutant were smaller than those from the wild-type (Fig 1D), having reduced width and thickness and, consequently, lower 1000-grain weights. Despite differences in spikelet numbers between the mutant and wild-type plants, no difference in SF was observed (Table 1). Many drooping leaf mutant genes encode members of the YABBY protein family and exhibit abnormal spikelets [24]. Unlike drooping leaf mutants previously reported, *dr* has normal spikelets.

**Table 1:** Agronomic traits of wild-type and *dr* mutant.

**Traits**	**PH (cm)**	**SL (cm)**	**PL (cm)**	**TP (No.)**	**PP (No.)**
Wild-type	93.2 ± 2.7	72.8 ± 3.3	20.4 ± 0.9	11.0 ± 2.1	11.0 ± 2.1
*dr* mutant	53.6 ± 2.3	38.0 ± 2.9	15.6 ± 1.1	7.4 ± 1.1	5.6 ± 0.5
Difference	***	***	***	*	***
**Traits**	**SP (No.)**	**SPP (No.)**	**SF (%)**	**TGW (G)**	**SPAD value**
Wild-type	136.2 ± 9.8	1507.0 ± 336.6	73.7 ± 6.1	24.6 ± 0.5	35.2 ± 2.0
*dr* mutant	46.3 ± 4.6	259.6 ± 41.1	66.8 ± 5.2	19.3 ± 0.5	21.0 ± 5.1
Difference	***	***	ns	***	**

PH; Plant Height, SL; Stem Length, PL; Panicle Length, TP; Tillers per Plant, PP; Panicles per plant, SP; Spikelets per Panicle, SPP; Spikelets per plant, SF; Spikelets fertility, TWG; Ten Grains Weight. SPAD value indicates the chlorophyll content of leaves. Asterisks indicate the significance of differences between the wild-type and *dr* mutant as determined by two tailed student’s t test (*, P<0.05; ***, p<0.001; ns, not significant).

The predominant characteristic of the *dr* mutant was the drooping leaf phenotype. The thick central midrib vein observed in wild-type leaf blades was diminished in the mutant leaves, being only half-formed and small in size (Fig 1E). Wild-type leaves were also darker green than mutant leaves (Fig 1F and 1I). Correspondingly, SPAD analysis revealed that chlorophyll content was almost 30% higher in wild-type than in the mutant leaves (Table 1). Leaves were then further characterized to establish the underlying cause of the drooping leaf phenotype in the *dr* mutant.

### Analysis of SiO_2_ abundance and its impact on the drooping leaf phenotype

Silica, which is deposited in leaves as the amorphous silica gel (SiO_2_) form, can act as a structural component to help maintain erect leaf morphology [25]. SiO_2_ abundance was assessed in wild-type and *dr* mutant to determine whether silica deposition contributed to the drooping leaf phenotype. As major plant nutrients such as nitrogen (N), phosphorus (P), and potassium (K) play significant roles in plant growth and development, N, P, and K were also assessed in wild-type and mutant leaves, stems, and roots at the maximum tillering stage.

**Fig 2:**
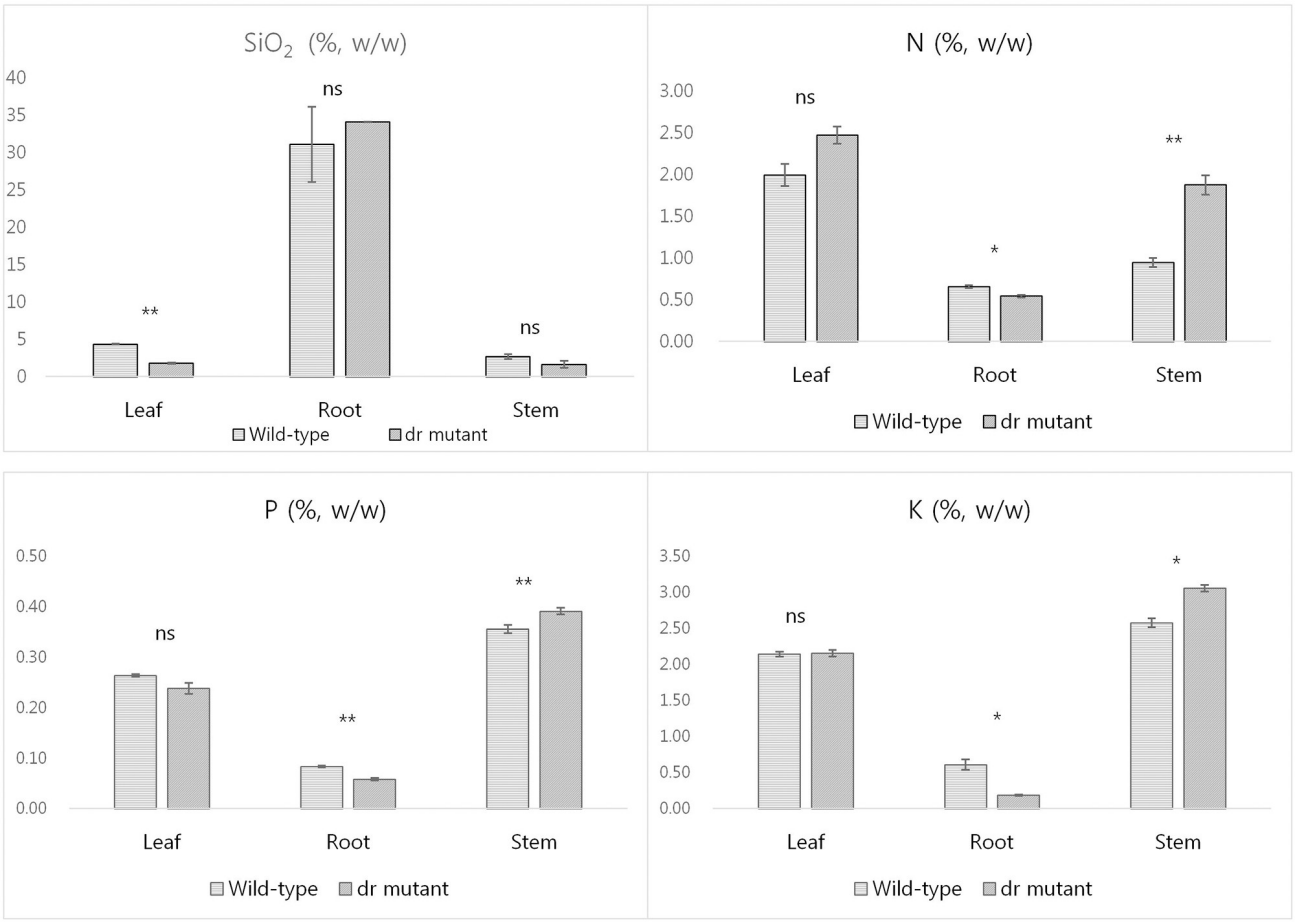
SiO_2_, N, P, and K content analysis in the wild-type and the *dr* mutant. Asterisks indicate the significance of differences between the wild-type and the *dr* mutant as determined by F-test (*, P<0.05; **, p<0.01; ns, not significant).

As shown in Fig 2, N, P, and K levels did not differ in leaves, but a significant difference was observed between wild-type and mutant stems and roots. By contrast, SiO_2_ levels were lower in mutant leaves than in wild-type leaves, but differences were not observed in other plant parts. Of the components assessed, only SiO_2_ exhibited reduced abundance in *dr* mutant leaves compared with the wild-type. This confirmed that SiO_2_ accumulation was lower in the mutant than in wild-type leaves, suggesting that lower SiO_2_ abundance might be responsible for the drooping leaf phenotype (S2 Table).

### Histological analysis of the *dr* mutant leaves

In rice leaves, silica is primarily deposited in epidermal cell walls beneath the thin cuticle layer: this is termed the cuticle-silica double layer [26–29]. Two types of silicified cells are found in rice leaf blades: dumbbell-like shaped silica cells and silica bodies derived from motor cells [30, 31].

**Fig 3:**
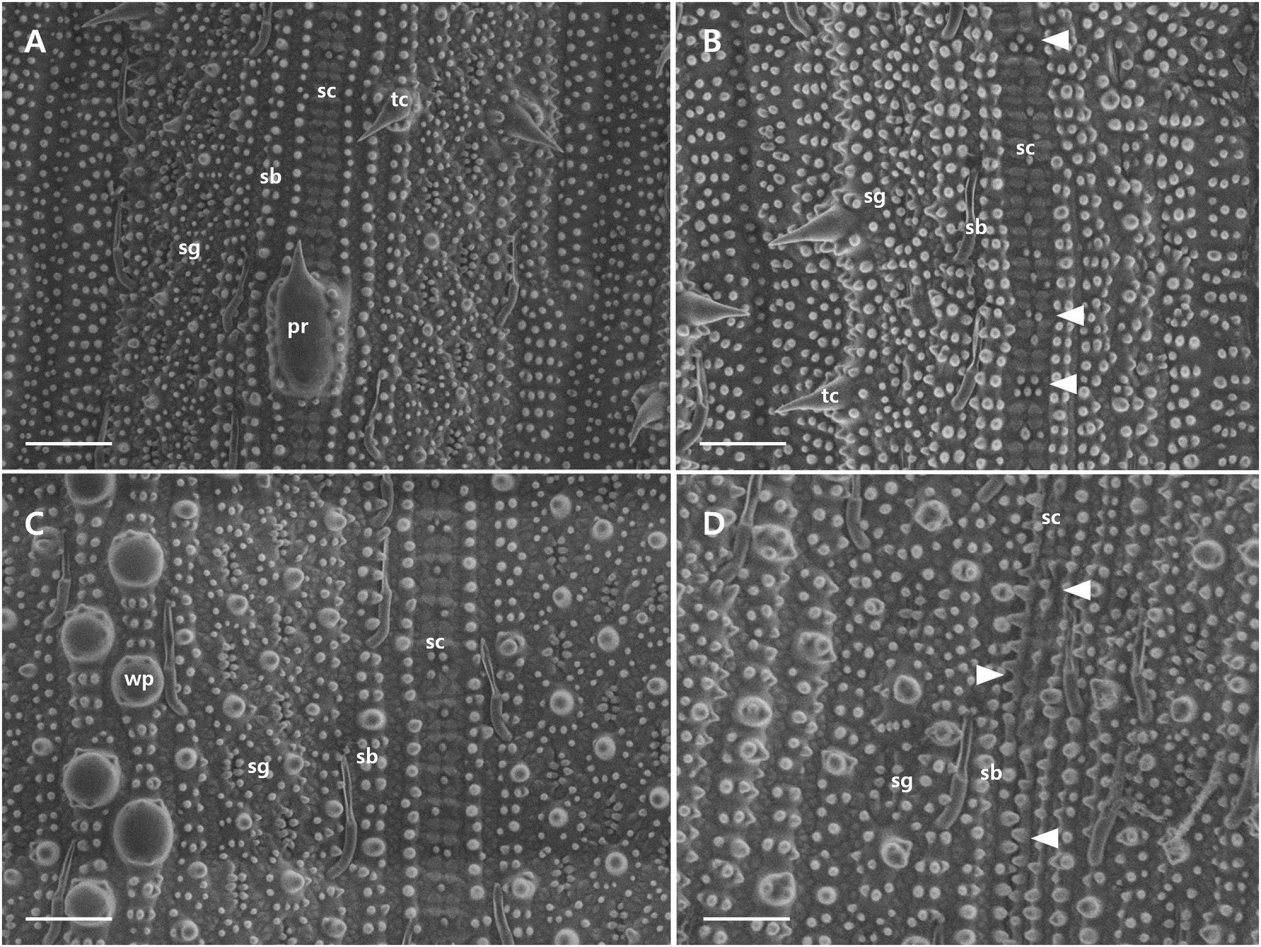
Scanning Electron Micrograph (SEM) of leaf epidermis (1000× magnification). (A, B) The adaxial surface in the wild-type and the *dr* mutant (C, D). The adaxial surface in the wild-type and the *dr* mutant. sb; silica body, sc; silicified dumbbell-shaped cells, sg; stomatal guard, pr; protuberances, tc; trichome, wp; wart-like protuberance. Arrow heads indicate undeveloped or degraded silica cells. Bars = 50 μm.

SEM was used to examine surface cells from leaves at the maximum tillering stage. Silica composition differed significantly between wild-type and the mutant leaves. On the adaxial side, wild-type leaves exhibited staunched silicified dumbbell-shaped cells along the vein, but mutant leaves had disordered and undeveloped cells (Fig 3A and 3B). Uniform silicified dumbbell-shaped cells were also found at the abaxial surface of wild-type leaves, whereas mutant leaves displayed degraded silica cells (Fig 3C and 3D).

**Fig 4:**
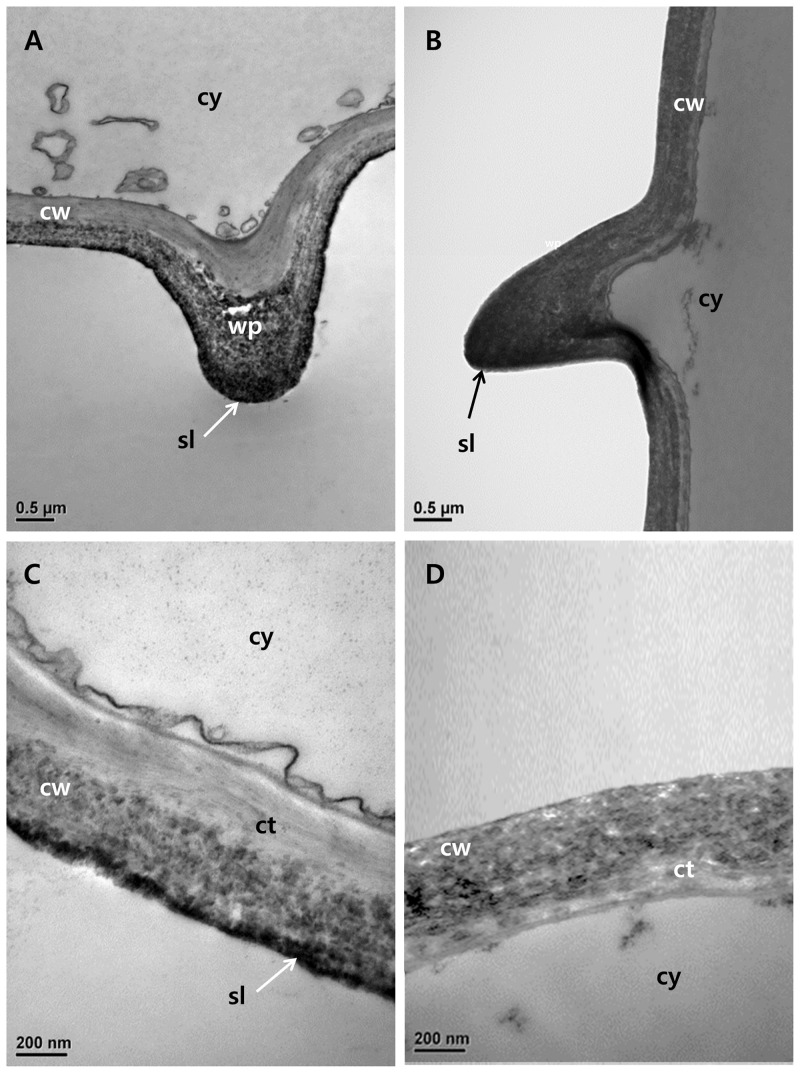
Analysis of leaf structures by transmission electron microscopy (TEM). (A) Epidermal cell wall of the wild-type in the wp region. (B) Epidermal cell wall of the *dr* mutant in the wp region. (C) Epidermal cell wall of the wild-type without the wp region. (D) Epidermal cell wall of the *dr* mutant without the wp region. Cy; cytoplasm, cw; cell wall, sl; silicon layer, wp; wart-like protuberances, ct; cuticle layer. Bars = 0.5 μm.

TEM was used to examine leaf cuticle membranes. Wild-type epidermis cells exhibited wart-like protuberances with electron-dense silica regions, but these structures were ambiguous in the mutant cells (Fig 4A and 4B). Wild-type epidermis cells without wart-like protuberances also had electron-dense silica layers, but these were not observed in *dr* mutant cells (Fig 4C and 4D). Cell walls, cuticles, and silica layers, were much thicker in wild-type than in mutant leaves, confirming that the *dr* mutant accumulated less silica than wild-type plants.

**Fig 5:**
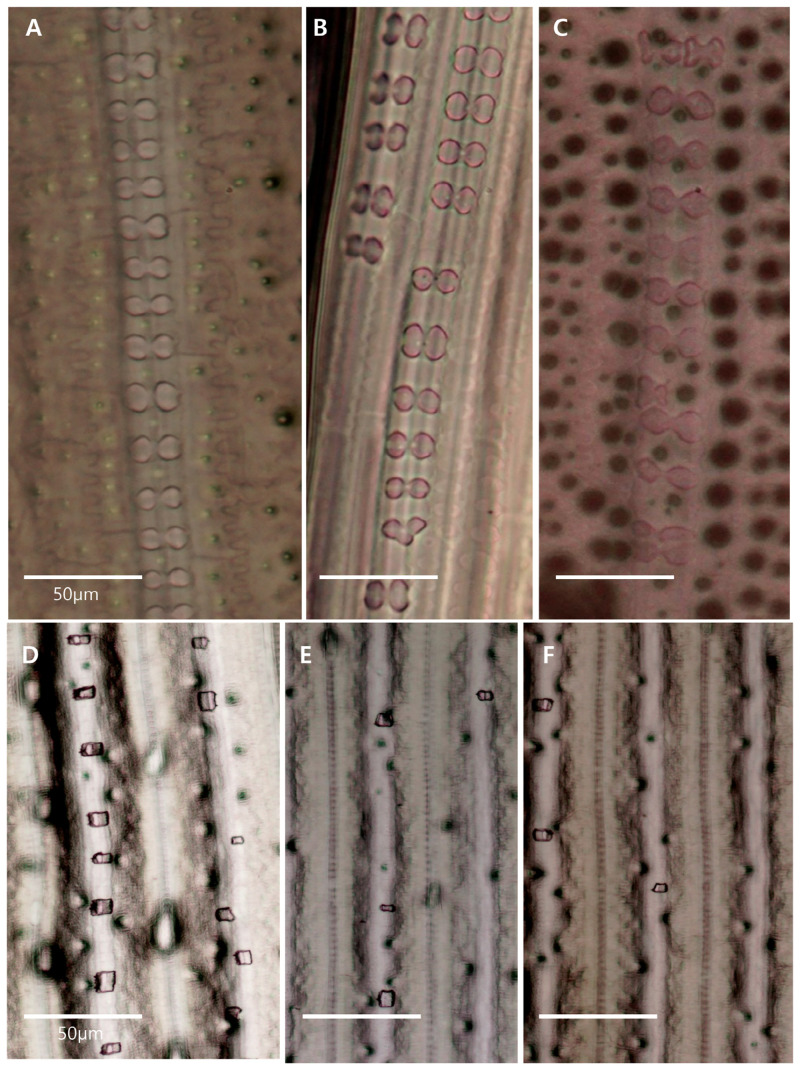
Optical microscopic images of leaves and motor cells (400× magnification, upper; 100×, lower). (A, D) wild-type. (B, E) RNAi plant. (C, F) *dr* mutant. Bars = 50 μm.

Silica deposition pattern of the leaves in the secondary cell wall was examined using optical microscopy with a phenol-safranin staining method. In wild-type leaves, silicified dumbbell-shaped cells were arranged in a uniform manner along the vein and were opaque with silica (Fig 5A). By contrast, dumbbell-shaped cells in RNAi and *dr* leaves were transparent and had irregular leaf shapes (Fig 5B and 5C). Motor cell size and density was also reduced in RNAi and *dr* leaves (Fig 5E and 5F) compared with wild-type leaves (Fig 5D).

### Genetic analysis and fine mapping of the *dr* mutation

F_1_ and F_2_ populations from crosses between the *dr* mutant and Ilpum lines were used to determine the inheritance of the *dr* trait. All F_1_ plants exhibited the wild-type phenotype, indicating that the *dr* trait was recessive. The F_2_ segregating population divided into two groups: *dr*-type and wild-type. Of the 250 F_2_ plants examined, 201 exhibited the wild-type phenotype and 49 exhibited the *dr* phenotype, consistent with the expected 3:1 Mendelian segregation ratio (Table 2). These results suggest that the *dr* trait was controlled by a single recessive gene.

**Table 2:** Genetic segregation of the drooping leaf trait in F_1_ and F_2_ populations derived from a cross between the wild-type and *dr* mutant (χ^2^_0.05(1)_ = 3.841).

Cross combination	Generation	No. of plant		Total	χ2 (3:1)	p-Value
		Wild-type	*dr* mutant			
*dr* mutant / Ilpum	F_1_	35	0	35	-	-
	F_2_	104	26	130	1.7333	0.188
	F_2_	97	23	120	2.1778	0.14

**Fig 6:**
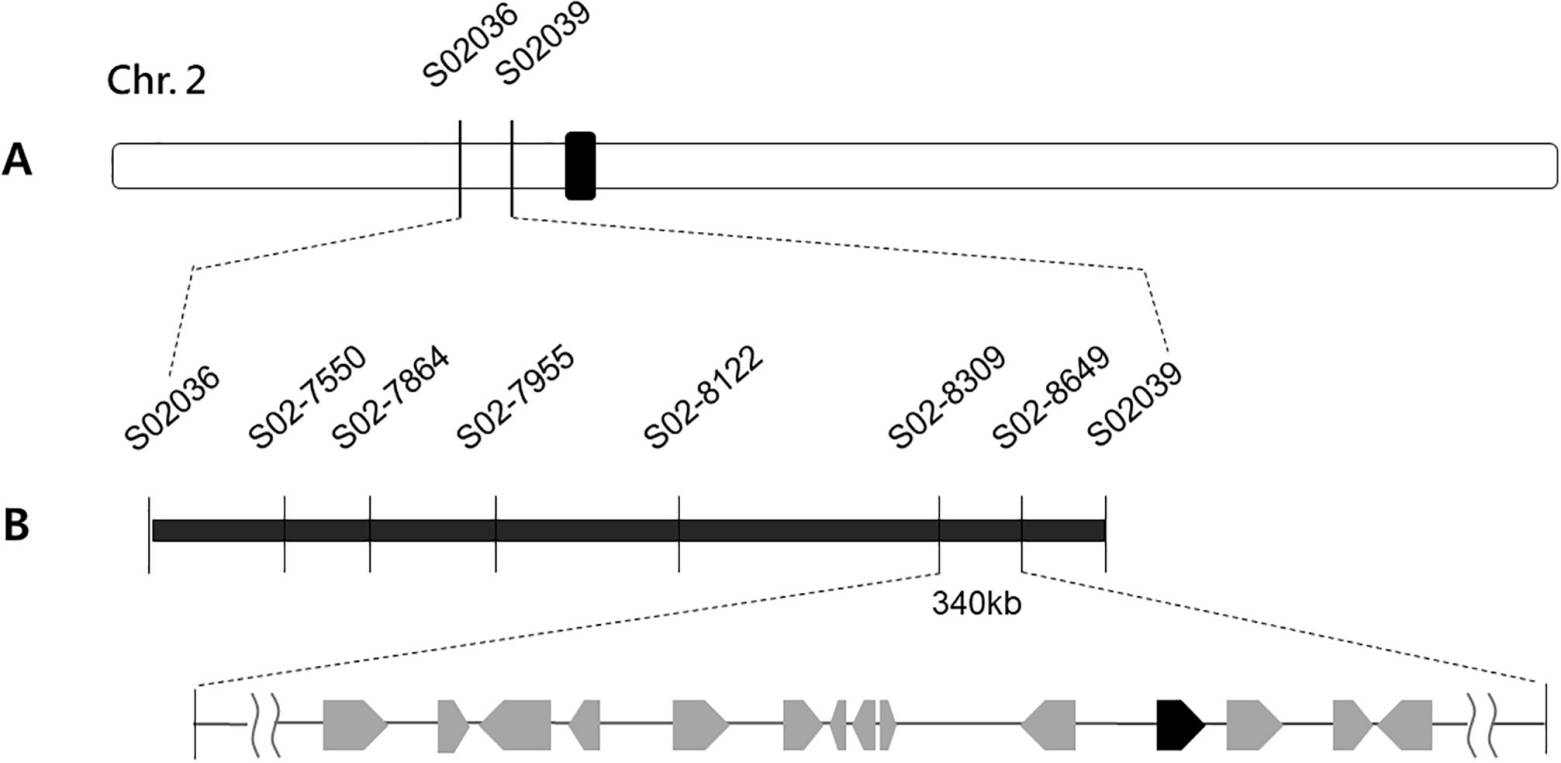
Positional cloning of the *dr* gene. (A) Genetic mapping of the *dr* locus with STS markers. (B) Fine-mapping of the *dr* locus with additional STS markers. The candidate gene was mapped to a 340kb region on chromosome 2.

To determine the physical location of the dr gene, F_2_ plants derived from cross between Milyang23 and dr mutant were used for mapping. BSA using 98 STS markers revealed that two markers, S02036 and S02039, located on the short arm of chromosome 2, showed significant polymorphisms between the wild-type and *dr* bulk samples (Fig 6A). Additional STS markers adjacent to the flanking markers were used to further refine the region to 340 kb between the S02-8309 and S02-8649 markers. Forty-five candidate genes within this region were identified using the RAP-DB database (version IRGSP 1.0; Fig 6B).

### Analysis of the candidate gene

**Fig 7:**
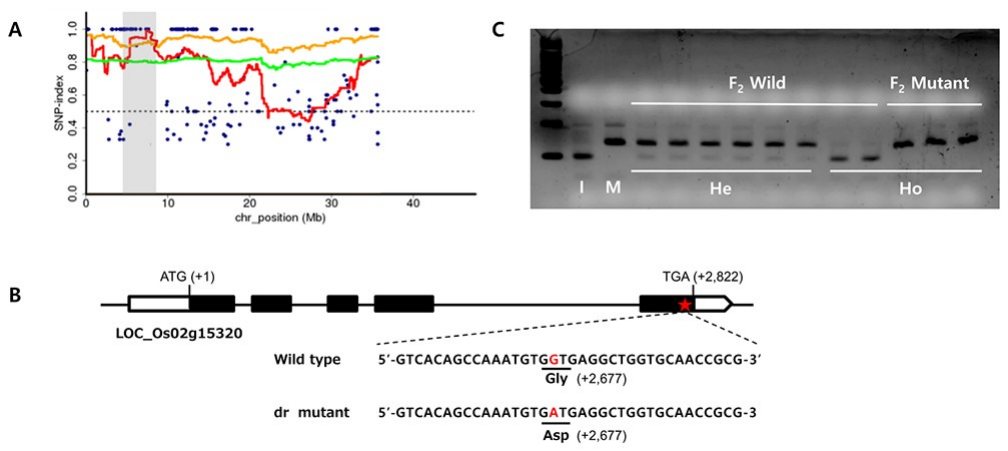
Identification of the *dr* gene. (A) SNP index plot of chr. 2 for identification of genomic regions harboring the causal mutation of *dr* gene. Gray shaded area is the region of the casual mutation. Blue dots represent SNP index values at a SNP position. Red line represents the sliding window average of SNP index values of the 4Mb interval with 10kb increments. Green line indicates the sliding window average of 95% confidence interval. Orange line indicates the sliding window average of 99% confidence interval. (B) Schematic diagram of the *dr* gene. Black rectangles represent exons and the red star represents the mutation site. (C) Co-segregation analysis of F_2_ plants using dCAPs markers. I; Ilpum, *dr*; *dr*, He; Heterozygous, Ho; Homozygous.

To locate the mutation and the gene responsible for the drooping leaf trait, a bulk population (25 plants) of DNA from F_2_ plants derived from a cross between *dr* mutant and Ilpum were used for whole genome sequencing and MutMap analysis. The sequence reads were aligned to the reference, Ilpum sequence. The SNP index of each SNP position was calculated, and SNP index plots were generated. According to the average SNP index peak, the most probable candidate region was detected in the 7–9 Mb region of chromosome 2 (Fig 7A) and it means that site is the most probable candidate region.

Thirty-four SNPs with an SNP index of 1 were found in the 340 kb candidate region between markers S02-8309 and S02-8649. Five of these SNPs were located in genic regions. DNA sequencing of each of the candidate genes was conducted to identify the gene associated with the *dr* phenotype. Comparisons between the *dr* mutant and wild-type sequences revealed a single point mutation in the LOC_Os02g15230 gene, which encodes GDSL esterase. The point mutation occurred in the fifth exon, at Open reading frame (ORF) nucleotide 2,677. The mutation substituted guanine with adenine, resulting in an amino acid change from glycine (Gly) to aspartic acid (Asp) in the *dr* mutant (Fig 7B). Co-segregation analysis of this SNP was performed using derived cleaved amplified polymorphic sequence (dCAPs) marker, and complete co-segregation of genotypes and phenotypes was observed in the F_2_ population (Fig 7C).

### Validation of the mutation causing the *dr* phenotype

**Fig 8:**
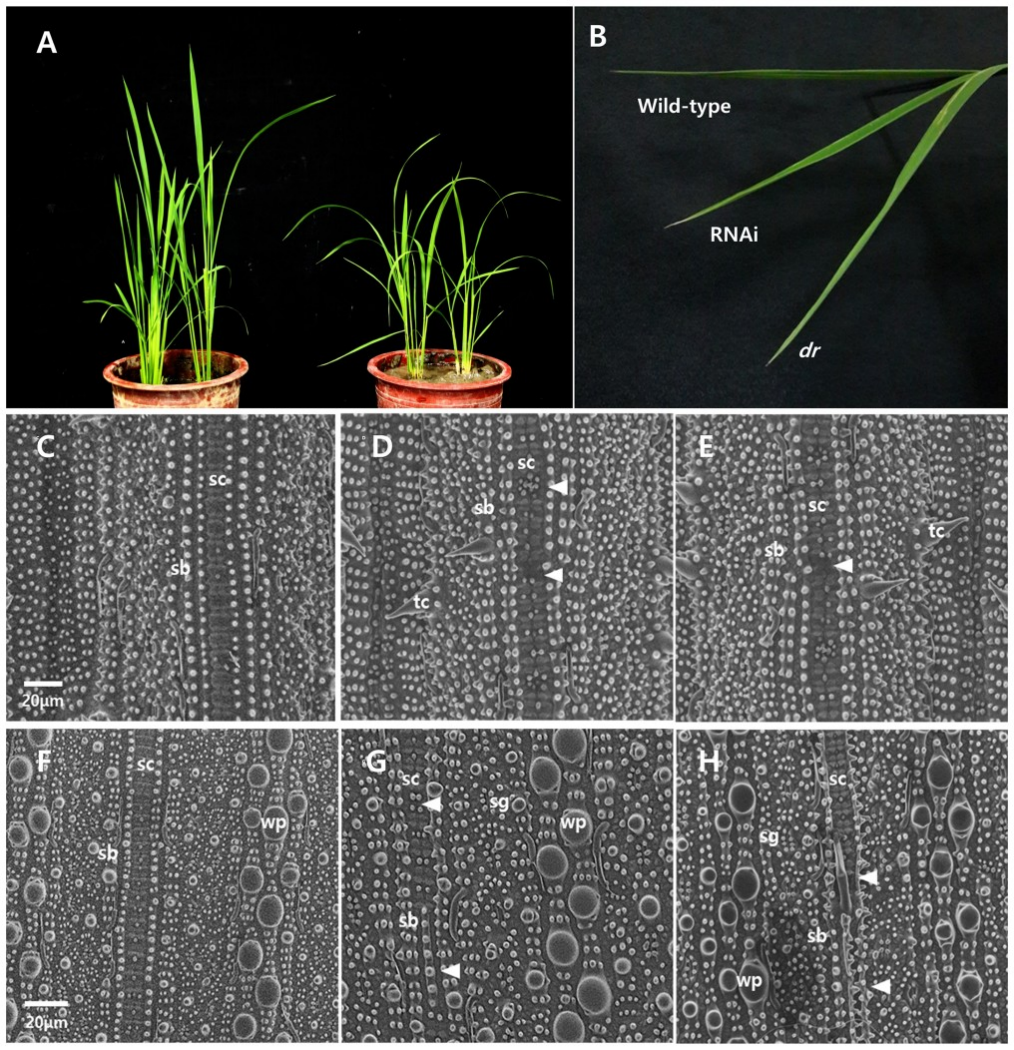
Comparison of the wild-type, *dr* mutant, and the RNAi plant leaf epidermal phenotypes using SEM (100× magnification). (A) Overall shape of plants on 60-day-old in the wild-type (left) and the RNAi (right). (B) Comparison leaf shape in the wild-type, RNAi and the *dr* mutant. (C, D, E) The adaxial surface in the wild-type, RNAi and the *dr* mutant. (F, G, H) The adaxial surface in the wild-type, RNAi and the *dr* mutant. sb; silica body, sc; silicified dumbbell-shaped cells, sg; stomatal guard, pr; protuberances, tc; trichome, wp; wart-like protuberance. Arrow heads indicate undeveloped or degraded silica cells. Bars = 20 μm.

To verify whether the LOC_Os02g15230 gene was responsible for the drooping leaf phenotype, dsRNA-mediated interference (RNAi) transgenic plants targeting the candidate gene were developed. RNAi expression partially induced the drooping leaf phenotype (Fig 8A and 8B). RNAi plants also exhibited similar silica distribution to the *dr* mutant. SEM visualization revealed that both RNAi plants and the *dr* mutant had several undeveloped and degraded silicified dumbbell-shaped cells at the adaxial and abaxial sides of the leaf, unlike wild-type leaves (Fig 8C–8H). Silica development and degradation in RNAi plants was at intermediate levels between wild-type and the *dr* mutant, consistent with the leaf shape observations. These results demonstrated that the mutant phenotype resulted from loss-of-function of the *dr* gene.

### *DR* gene encoding a GDSL esterase/lipase

Database analysis suggested that the *DR* gene is a member of the GDSL esterase/lipase family, which is containing a SGNH hydrolase domain. The GDSL esterase/lipase has four important catalytic residues Ser-Gly-Asn-His in the conserved blocks I, II, III and V [32]. The *DR* gene also has these residues and it indicates that the *DR* gene may have esterase/lipase activity. GDSL esterase/lipases are involved in the regulation of plant development, morphogenesis, and synthesis of secondary metabolites [33, 34].

**Fig 9:**
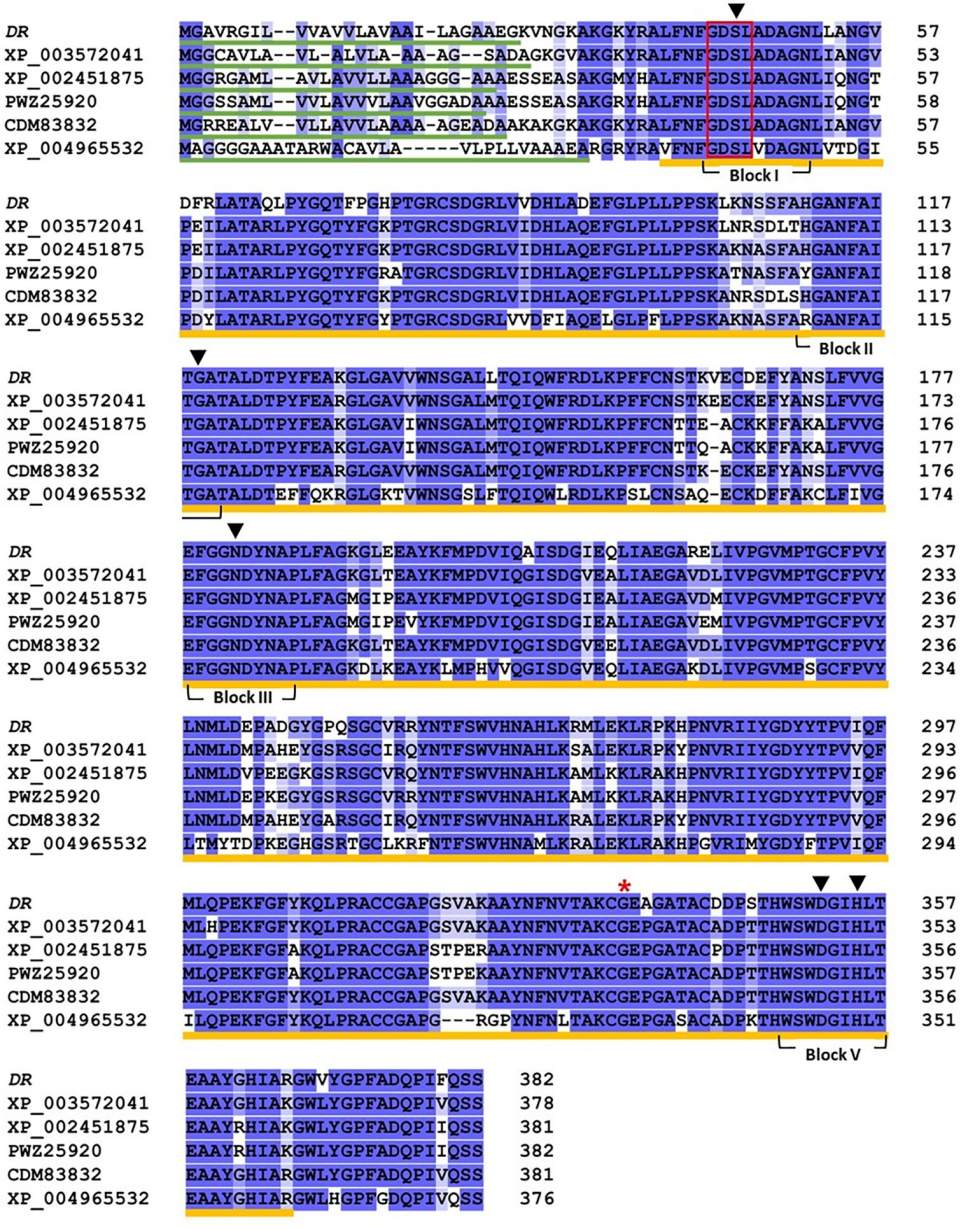
Multiple sequences alignment of the *DR* protein in various crops. The backgournd color (dark blue and light blue) boxes represent identical or similar amino acids. Block I to V indicate the conserved domains. N-terminal signal peptides and SGNH hydrolase domains are underlined with green and yellow line. GDSL motif is surrounded by red box. Red asterisks indicate amino acid residue which is changed by missense mutation in *dr* gene. The black arrowheads mark indicate the catalytic residues. XP_003572041; *Brachypodium distachyon*, XP_002451875; *Sorghum bicolor*, PWZ25920; *Zea mays*, CDM83832; *Triticum aestivum*, XP_004965532; *Setaria italica*.

Multiple sequence alignment showed that the *DR* gene has highly conserved domain and motif within its amino acid sequences. Orthologs of the rice *DR* gene were found in several cereal crops, including *Brachypodium distachyon* (XP_003572041; 88% amino acid indentity), *Sorghum bicolor* (XP_002451875; 83% amino acid indentity), *Zea mays* (PWZ25920; 83% amino acid indentity), *Triticum aestivum* (CDM83832; 88% amino acid indentity), *Setaria italica* (XP_004965532; 73% amino acid indentity) (Fig 9).

## Discussion

During photosynthesis, plants trap light energy with their leaves. Properly curved leaves receive optimum light and increase yield [35]. Although leaf architecture-related genes have been studied for several decades, the mechanisms regulating leaf architecture remain poorly understood [36]. In this study, we isolated the *dr* gene and determined the cause of the drooping leaf phenotype.

Map-based approach and resequencing data from MutMap-assisted SNP genotyping revealed that the *DR* gene was LOC_Os02g15230 on chromosome 2, and the causative SNP for the mutant was located in the 5^th^ exon of the gene (Fig 6). RNAi-transgenic plants exhibited the same phenotypes as the *dr* mutant, confirming that *DR* gene was LOC_Os02g15230 (Fig 8).

Previously, another mutant in LOC_Os02g15230, namely, brittle leaf sheath1 (*bs1*), was identified [37]. This is a natural mutant from Nipponbare in which a single base at the second exon-intron junction caused an unspliced intron and premature stop codon and consequently resulted in brittle leaf sheath phenotype. In fact, although Zhang et al. [37] didn’t mention about the drooping leaf phenotype and didn’t observe abnormal silica deposition in leaves as well in the *bs1* mutant, its appearance looks similar to the *dr* mutant. On the other hand, the *dr* mutant in this study didn’t exhibit brittle stem phenotypes. This indicates that *dr* and *bs1* are different alleles of LOC_Os02g15230 showing a little different phenotypes, which yet to be tested for allelism with functional studies.

In general, silica deposition enhances strength and rigidity by reinforcing plant cell walls, and erect leaf blade structure is compromised when silica levels are reduced [38, 39]. Previous studies revealed that the leaves without silica showed droopy and less rigid surface [40–42]. SEM and TEM analysis indicated that *dr* leaves contained fewer silicified dumbbell-shaped cells and had a thinner silica layer than wild-type leaves (Figs 3 and 4). Plant nutrient analysis confirmed that silica accumulation was significantly reduced in *dr* leaves compared with the wild-type (Fig 2), suggesting that insufficient silica accumulation resulted in the drooping leaf phenotype. The mechanism of abnormal Si deposition in the mutant leaves is yet to be studied in relation to the report by Zhang et al. [37] that a natural mutation of the LOC_Os02g15230 showed impaired secondary wall pattern and abnormal pleotropic phenotypes such as brittle leaf sheath, abnormal xylem, and reduced growth, although phenotypes of *bs1* and *dr* mutants are controlled by different alleles and different genetic backgrounds as well.

This study clarifies the relationship between the *dr* phenotype and silica abundance and demonstrates that the *dr* gene plays an important role in determining the shape of rice leaves. These results advance our understanding of the mechanisms underlying leaf morphology and provide important information for rice breeding programs.

## Supporting information

S1 Table Primer information used for mapping. (DOCX)

S2 Table Abundance of SiO_2_ and other plant nutrients in leaf, stem, and root tissues of wild-type and *dr* mutant. (DOCX)
