# Supplementary material for: The DROOPING LEAF (DR) gene encoding GDSL esterase is involved in silica deposition in rice (Oryza sativa L.)
Source: PLoS One. 2020 Sep 10;15(9):e0238887. doi: 10.1371/journal.pone.0238887 (PMC7482962; doi:10.1371/journal.pone.0238887)
Supplement: S1 Table — (DOCX) [file pone.0238887.s001.docx]

| **Primer name** | **Forward primer sequence (5'→'3')** | **Reverse primer sequence (5'→3')** |
| --- | --- | --- |
| S02036 | TAAGCACCATGAATCCGTGA | ATGGTCAACCGGTGTGCT |
| S02039 | CGCAAACAACATTCCAAAAA | CAGCGGCATTCGTCTAAAA |
| S02-7550 | AACTTTGAGGCTGCATTAATTTG | GGATAGATTCCACCGCAAG |
| S02-7864 | CCGTTGCAAATGCAAATCTT | GTTTTTATGGGATGGAGGGG |
| S02-7906 | ACACACGCAAGACAACGAAA | TACCTCGACTAGCATCGCCT |
| S02-7955 | TAACGTATGTACACCGCCGA | TCAAATGAGTTGGGGGACAT |
| S02-8122 | AAGTGACATCAGGTTTCCACAA | TGAATACACGGAGAGAGCGA |
| S02-8309 | GTACTTGCGATTGTGCCTGA | GGTCACAACTCACAACACACACT |
| S02-8479 | ATCACCGGCATGGTCTATTC | TTTAGCATGCACTGGTCAGC |
| S02-8649 | GTCCTCTCGACGGATGAAAA | CAAACATTTTTAACTTTGACCATTG |

S1 Table. Primer information used for mapping.
