# Supplementary material for: The DROOPING LEAF (DR) gene encoding GDSL esterase is involved in silica deposition in rice (Oryza sativa L.)
Source: PLoS One. 2020 Sep 10;15(9):e0238887. doi: 10.1371/journal.pone.0238887 (PMC7482962; doi:10.1371/journal.pone.0238887)
Supplement: S2 Table — (DOCX) [file pone.0238887.s002.docx]

| **Component** | **Type** | **Part** | **Amount (%)** | **SE** | **F-test** |
| --- | --- | --- | --- | --- | --- |
| SiO_2_ | *dr* mutant | leaf | 1.79 ± 0.18 | 0.10 | *** |
|  | Wild-type |  | 4.33 ± 0.06 | 0.04 |  |
|  | *dr* mutant | stem | 1.63 ± 0.92 | 0.53 | ns |
|  | Wild-type |  | 2.67 ± 0.33 | 0.19 |  |
|  | *dr* mutant | root | 34.08 ± 0.03 | 0.02 | ns |
|  | Wild-type |  | 31.08 ± 5.09 | 2.91 |  |
| N | *dr* mutant | leaf | 2.47 ± 0.21 | 0.15 | ns |
|  | Wild-type |  | 2.00 ± 0.26 | 0.15 |  |
|  | *dr* mutant | stem | 1.87 ± 0.23 | 0.13 | ** |
|  | Wild-type |  | 0.94 ± 0.11 | 0.06 |  |
|  | *dr* mutant | root | 0.54 ± 0.03 | 0.02 | * |
|  | Wild-type |  | 0.66 ± 0.03 | 0.02 |  |
| P | *dr* mutant | leaf | 0.24 ± 0.02 | 0.01 | ns |
|  | Wild-type |  | 0.26 ± 0.01 | 0.01 |  |
|  | *dr* mutant | stem | 0.39 ± 0.01 | 0.01 | ** |
|  | Wild-type |  | 0.36 ± 0.02 | 0.01 |  |
|  | *dr* mutant | root | 0.06 ± 0.01 | 0.01 | ** |
|  | Wild-type |  | 0.08 ± 0.01 | 0.01 |  |
| K | *dr* mutant | leaf | 2.15 ± 0.09 | 0.05 | ns |
|  | Wild-type |  | 2.14 ± 0.06 | 0.04 |  |
|  | *dr* mutant | stem | 3.06 ± 0.10 | 0.05 | * |
|  | Wild-type |  | 2.58 ± 0.12 | 0.07 |  |
|  | *dr* mutant | root | 0.18 ± 0.02 | 0.01 | * |
|  | Wild-type |  | 0.61 ± 0.14 | 0.08 |  |

S2 Table. Abundance of SiO_2_ and other nutrients in leaf, stem, and root tissues of the wild-type and *dr* mutant.
